# Nonlinear physics opens a new paradigm for accurate transcription start site prediction

**DOI:** 10.1186/s12859-022-05129-4

**Published:** 2022-12-30

**Authors:** José Antonio Barbero-Aparicio, Santiago Cuesta-Lopez, César Ignacio García-Osorio, Javier Pérez-Rodríguez, Nicolás García-Pedrajas

**Affiliations:** 1 Departamento de Informática, Universidad de Burgos, Avda. de Cantabria s/n, 09006 Burgos, Spain; 2 Universidad de Burgos, Hospital del Rey, s/n, 09001 Burgos, Spain; 3 ICAMCyL Foundation, Internacional Center for Advanced Materials and Raw Materials of Castilla y León, León Technology Park, main building, first floor, offices 106-108, C/Julia Morros s/n, Armunia, 24009 León, Spain; 4 Departamento de Métodos Cuantitativos, Universidad de Loyola Andalucía, Escritor Castilla Aguayo, 4, 14004 Córdoba, Spain; 5 Department of Computing and Numerical Analysis, University of Córdoba, Edificio Albert Einstein, Campus de Rabanales, 14071 Córdoba, Spain

**Keywords:** DNA modelling, DNA breathing, Machine Learning, TSS prediction, SVM, String kernels

## Abstract

There is evidence that DNA breathing (spontaneous opening of the DNA strands) plays a relevant role in the interactions of DNA with other molecules, and in particular in the transcription process. Therefore, having physical models that can predict these openings is of interest. However, this source of information has not been used before either in transcription start sites (TSSs) or promoter prediction. In this article, one such model is used as an additional information source that, when used by a machine learning (ML) model, improves the results of current methods for the prediction of TSSs. In addition, we provide evidence on the validity of the physical model, as it is able by itself to predict TSSs with high accuracy. This opens an exciting avenue of research at the intersection of statistical mechanics and ML, where ML models in bioinformatics can be improved using physical models of DNA as feature extractors.

## Background

Understanding DNA structure and dynamics is one of the most challenging subjects in biophysics. In the case of DNA, unraveling the processes by which specific binding sites are recognised by proteins, drugs, mutagens, and other molecules represents a fundamental step toward understanding its biological activity. The description by Watson and Crick of the double helix structure of DNA [1], thanks to the works of Rosalind Franklin, originally led to a fundamental postulate that “form is function” for biological molecules, meaning that the properties of the molecule are due only to its structure. Today, this vision has changed, and it is now understood that the dynamics of its base pairs are also essential for its function [2]. Even in the absence of enzymes involved in the reading or duplication of the code, DNA undergoes large amplitude fluctuations.

Indeed, the DNA double helix is a very dynamic molecule. The two strands of DNA are linked by hydrogen bonds (one of the weakest molecular bonds) at each of their bases, a double bond between the adenine and thymine bases, and a triple bond between the cytosine and guanine bases (which makes this second pair more difficult to separate than the first). These bonds can be broken due to thermal fluctuations, in one base or in several adjacent bases, forming a *bubble*. The opening of the DNA can therefore occur spontaneously (more likely the higher the temperature). This process is known as *DNA breathing* (or at high temperatures, as *DNA denaturation* or *melting*). There is increased evidence that bubbles affect molecular activity [3–7] and play an important role in the processes of *transcription* (the information contained in a portion of DNA is transmitted to an RNA strand) and *replication* (copying of DNA during cell division).

For this reason, several physical models of DNA have been proposed that try to model the behaviour of DNA and the dynamic processes of bubble creation and denaturation. Perhaps the most cited in the literature is the Peyrard-Bishop-Dauxois (PBD) model [8–11], a relatively simple one-dimensional model from which several variants have emerged, for example, including a potential barrier, which improves the description of bubbles [12]; adding particles with Brownian motion to simulate the behaviour of an enzyme and its interaction with DNA [13]; or adding a stacking term to incorporate the interaction between adjacent base pairs into the model, making it sequence dependent [14].

The kinetics of base pair opening dynamics has been well described following the exchange of protons from imino groups with water [15], showing that particular regions of the helix open by the action of thermal fluctuations, and suggesting the importance of sequence effects in the opening of particular tracks [16]. The lifetime of a base pair, i.e. the time during which it stays closed, is only of the order of a few milliseconds. Experiments show that these fluctuations, known by biologists as the *breathing* of DNA, are highly localised and may open a single base pair, while the adjacent ones stay closed [16–19].

### Denaturation bubbles

At the dawn of the twenty-first century, different studies examined the statistical and dynamical properties of *denaturation bubbles*, demonstrating their role in the melting transition of short oligomers and triggering strong debate in the statistical physics and biophysical communities [10, 20–25]. These studies pointed out the relationship of statistical properties and dynamical phenomena with respect to biological function and molecular mechanisms.

In this context, a series of studies initiated in 2004 by C.H. Choi, G. Kalosakas and K.O. Rasmussen [4, 6] raised a very interesting question within the community: *Is DNA, thanks to thermal aided structural fluctuations, capable of directing its own transcription initiation?*

Inspired by these findings, different works using the well-known Peyrard-Bishop-Dauxois (PBD) model [26] demonstrated both experimentally and theoretically that fluctuations play a much larger role in the flexibility of the molecule and in the function of DNA than was generally thought [3, 11, 27]. These results clearly showed that the PBD model can provide interesting information related to biological function and biological features at many levels. However, almost 20 years later, the question has not been fully answered despite various efforts, theoretical models, and experiments carried out by leading groups in the field. Moreover, some critical views and controversies are being debated [10, 20–25].

### Previous trials of PBD as a bioinformatic tool in the community

In this paper, we address this key challenge using a transversal approach. We examine bioinformatics and advanced classifiers methodologies using the statistical mechanics information from a theoretical approach: the PBD model, which provides valuable information about the structural stability and flexibility of DNA. We used our approach to explore massive ensembles of sequences from bioinformatics databases to try to detect transcription start sites (TSSs), benchmarking the results with data ensembles with experimentally confirmed TSSs. TSS recognition is one of the most challenging recognition problems in DNA sequences. The increasing amount of biological data accumulated in the last decades, together with the emergence of methods such as CAGE [28], has led to the development of numerous tools that can process these large amounts of data. However, unlike other problems such as translation initiation site (TIS) prediction, which can be considered solved with the latest prediction results [29], the problem of TSS detection remains a challenge even for state-of-the-art tools. This is due to its increased complexity, mainly because more than one TSS may exist in a single promoter. The lack of sequences including TSS information, as most known sequences in the databases are downstream of the TSS, also makes the recognition of TSSs more difficult than the recognition of other functional sites. Thus, many TSS recognisers have been developed [30, 31]. These programs usually exploit the features that differentiate TSSs from other genomic DNA. Despite the arduousness of this problem, locating the TSS means obtaining relevant information toward an understanding of transcription regulatory networks and in-silico findings of new genes.

Current achievements in bioinformatic methods for the detection of TSS use several approaches based on machine learning [32] and other techniques that work with sequence features [33]. Although some of them have tried to include additional information from sequences into the model using techniques like multiple sequence alignments [34], none have included statistical information of bubbles and openings. Although current methods for similar problems as TIS [29, 35, 36] or promoter identification [37] tend to use deep learning methods, in TSS prediction, we find that support vector machines (SVMs) are the most popular approach for this problem [31, 34, 38, 39].

We obtained two highly promising results. First, we showed that by using either base opening or bubble information, we can reach a classification performance for TSS detection with an accuracy similar to the methods using the base pair sequence. With this, we showed that by adding base pair opening and bubble information to classifiers, we changed the paradigm of automatic detection of TSS, providing a new source of information for the task. Second, we proved that by combining this new source with the standard of using the base pair sequence, we can obtain classifiers that are better than the best state-of-the-art methods.

## Results

### Models and parameter settings

Previous papers [31, 40–45] indicated that SVMs with string kernels are the best option to recognise functional sites in DNA sequences. Thus, we performed a preliminary study of the available methods that included position weight matrices, decision trees, *k*-nearest neighbours and SVMs with linear and Gaussian kernels and three different string kernels: locality improved kernel (LI) [40], weighted degree kernel (WD) and weighted degree kernel with shifts [44] (WDS). SVMs with the WD kernel consistently obtained the best results, and thus were chosen as the method to be compared with our proposal. This matches the approaches of previous works [46, 47]. WDS obtained marginally better results than WD, but with far higher computational complexity. We will refer throughout the paper to the SVM with WD kernels as SVMWD.

One of the problems with SVM classifiers is their sensitivity to parameter settings. Thus, we performed a parameter setting process each time an SVM was applied. We used 10-fold cross-validation and tested three window widths for the string kernel (6, 12 and 24) and several regularization values, $C = \{0.001, 0.01, 0.1, 1, 10, 100, 1\,000\}$, considering all 21 combinations. This parameter setting process was repeated each time an SVM was applied.

In contrast to other papers, where only a few sequences were tested, we used a realistic case of TSS recognition in which thousands of instances were evaluated. We tested our approach in the data set used to evaluate the ARTS [31] and RBF-TSS models [32], which can be downloaded from http://www.fml.tuebingen.mpg.de/raetsch/projects/arts. As explained in [31] and [32], the dataset is divided into three parts: training, validation and testing. As we did not use validation, this part was added to the training set. Training data set was extracted from dbTSS version 4, which is based on the UCSC human genome sequence assembly and annotation version 16 (“hg16”). This dataset contains transcription start sites of $12\,763$ RefSeq genes. RefSeq identifiers were extracted from dbTSSv4, and then the corresponding mRNA sequences using NCBI nucleotide batch retrieval were obtained. Next, these mRNAs were aligned to the hg16 genome using BLAT. From dbTSS, putative TSS positions were extracted and compared with the best alignment of the mRNA. Reference [31] discarded all positions that did not pass all the following checks: 1. chromosome and strand of the TSS position and of the best BLAT hit match; 2. TSS position was within 100 base pairs from the gene start, as found by the BLAT alignment; and 3. no already processed putative TSS was within 100bp of the current one. This procedure left $8\,508$ genes, each annotated with gene start and end.

To generate positive training data, windows of size $[-100, +100]$ around the TSS were extracted. To discriminatively train a classifier, one also needs to generate “negative” data. However, there is no single natural way of doing this. Since there are further yet unknown TSSs hidden in the rest of the genome, it is dangerous to sample negative points randomly from it. Thus, the researchers proceeded similarly to [48] by extracting “negative” points (again, windows of size $[-100, +100]$) from the interior of the gene. More precisely, they drew 10 negatives at random from locations between 100 bp downstream of the TSS and the end of the gene. Finally, $8\,508$ positive and $85\,042$ negative examples were obtained.

**Table 1:** Datasets summary

Dataset	Positive instances	Negative instances
hg16 (train)	$8\,508$	$85\,042$
hg17 (test)	$1\,024$	$10\,238$

To obtain the testing dataset, we again followed the setup of [31]. All “new” genes from dbTSSv5 [49] (which is based on hg17) for which a representative TSS was identified (i.e., the field “the selected representative TSS” is not empty) were taken. From dbTSSv5, all genes that already appeared in dbTSSv4 according to the RefSeq NM identifier were removed. To take care of cases where IDs changed over time or were not unique, all genes from dbTSSv5 for which mRNAs overlapped by more than 30% were also removed. Thus, a total of $1\,024$ TSSs remained to be used in a comparative evaluation. This left us with $1\,024$ positive testing samples and $10\,238$ negative testing samples. Table 1 summarizes the training and testing datasets.

To obtain the opening and bubble probabilities, the window size used was $[-150, 150]$. Thus, 300 bases are used in the PBD model. Of these 300 probabilities, the first 50 and the last 50 are discarded, leaving the 200 central probabilities of opening. Such probabilities have been removed to avoid noise or false information caused by the effects generated by the boundary conditions applied to solve the PBD model, as explained in previous studies [50].

One of the common features of most Bioinformatics problems is the class-imbalance nature of the datasets [47]. This problem appears when the number of positive instances of the dataset is clearly outnumbered by the number of negative instances. Usually, the degree of imbalance of the datasets is measured using the imbalance ratio (IR) of the negative class with respect to the positive one. In our dataset we have $8\,508$ positive and $85\,042$ negative examples, thus having an IR of 10:1, which can be considered high. Usually, the positive class is termed the minority class, whereas the negative class is termed the majority class.

Most learning algorithms are harmed in their performance if class-imbalance is not dealt with. A simple, yet effective, method is random undersampling [51]. In random undersampling, only a random subsample of the majority class is used for the learning process. The number of instances sampled from the majority class is fixed by a predefined desired IR. This is the method we have used in all our experiments. Random undersampling of the majority class was performed prior to any learning, with a final IR in the dataset of 1:1.

### Performance measures

To evaluate the obtained classifiers, we used the standard measures for imbalanced data. Given the number of true positives (TP), false positives (FP), true negatives (TN) and false negatives (FN), we used the sensitivity $Sn = \frac{ TP }{ TP + FN }$ and specificity $Sp = \frac{ TN }{ TN + FP }$. The geometric mean of these two measures, $G\text {-mean} = \sqrt{ Sp \cdot Sn }$, was our first classification metric. Many classifiers are subject to a threshold that can be varied to achieve different values of the above measures. For that type of classifier, receiver operating characteristic (ROC) curves can be constructed. A ROC curve is a graphical plot of the $TP _ rate$ (sensitivity) against the $FP _ rate$ ($1\ -$ specificity or $FP _ rate = \frac{ FP }{ TN + FP }$) of a binary classifier system as its discrimination threshold is varied. The perfect model achieves a true positive rate of 1 and false positive rate of 0. A random guess is represented by a line connecting the points (0, 0) and (1, 1). ROC curves are a good measure of the performance of the classifiers. Furthermore, from this curve, a new measure, area under the ROC curve (auROC), can be obtained. auROC is a very good overall measure for comparing algorithms and a useful metric for classifier performance, as it is independent of the decision criterion selected and prior probabilities.

We use the metrics *G*-mean and auROC because they provide two different views of the performance of the classifiers. The auROC values describe the general behaviour of the classifier. However, when used in practice, we must establish a threshold for the classification of a query pattern. *G*-mean provides the required snapshot of the classifier performance when we set the needed threshold.

### Effects of using bubble information

**Fig. 1:**
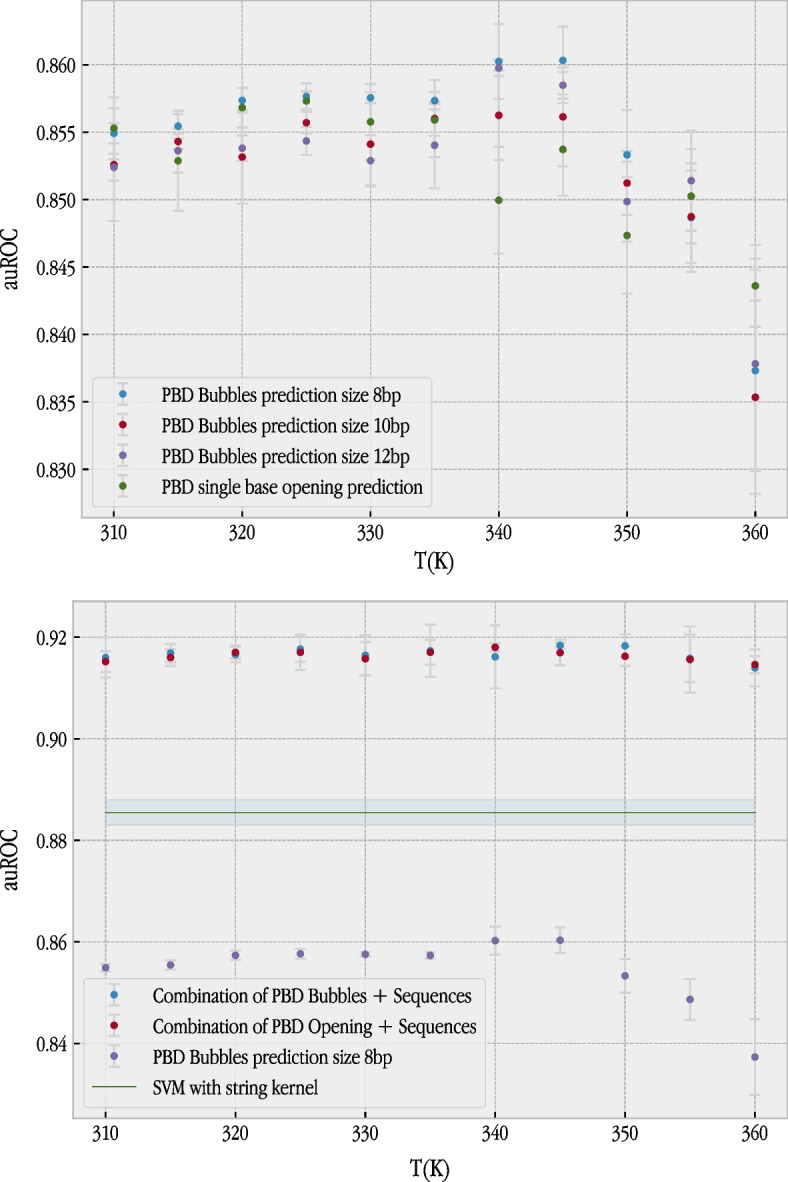
Top: Performance of our model evaluated over testing set after using information coded in PBD model analysis, using prediction of bubble occurrence probability of different sizes, or just single base pair opening probability. The information was evaluated as a function of temperature, showing a clear dependence of the information richness with respect to temperature variation. Bottom: The final results of the best models, obtained with the probabilities of bubbles of 8 bp and with the probabilities of opening in a single base (“bubbles” of size 1 bp)

**Table 2:** Results

Sequences of length 8 and different temperatures			Combination of sequences and bubbles using		
			a hybrid kernel and different temperatures		
Temperature	auROC	G-mean	Temperature	auROC	G-mean
310K	0.8549 ± 0.0007	0.7874 ± 0.0016	310K	0.9160 ± 0.0040	0.8541 ± 0.0062
315K	0.8554 ± 0.0009	0.7891 ± 0.0011	315K	0.9169 ± 0.0018	0.8567 ± 0.0051
320K	0.8574 ± 0.0009	0.7896 ± 0.0027	320K	0.9166 ± 0.0015	0.8535 ± 0.0160
325K	0.8577 ± 0.0010	0.7899 ± 0.0012	325K	0.9176 ± 0.0025	0.8540 ± 0.0027
330K	0.8575 ± 0.0004	0.7900 ± 0.0013	330K	0.9164 ± 0.0040	0.8555 ± 0.0072
335K	0.8573 ± 0.0007	0.7877 ± 0.0011	335K	0.9173 ± 0.0051	0.8543 ± 0.0052
340K	0.8602 ± 0.0028	0.7898 ± 0.0016	340K	0.9161 ± 0.0062	0.8534 ± 0.0156
345K	**0**.**8603** ± 0.0025	0.7925 ± 0.0014	345K	**0**.**9184** ± 0.0005	**0**.**8590** ± 0.0091
350K	0.8533 ± 0.0033	0.7901 ± 0.0017	350K	0.9183 ± 0.0023	0.8568 ± 0.0045
355K	0.8487 ± 0.0040	0.7955 ± 0.0012	355K	0.9158 ± 0.0047	0.8501 ± 0.0142
360K	0.8373 ± 0.0075	**0**.**8083** ± 0.0012	360K	0.9140 ± 0.0036	0.8452 ± 0.0059

The best values for each performance metric are highlighted in bold

Our first experiment established the performance of our baseline method. We performed experiments with the standard approach of using the sequence as the source of information, along with the SVMWD method. This experiment obtained a *G*-mean of 0.8066±0.0030 for a 95% confidence interval and an auROC of 0.8855±0.0025. Our second experiment validated the “bubble” models as a method for TSS recognition. We used SVMs with a Gaussian kernel, as the inputs are real values. Table 2 lists the results obtained for different temperatures (also represented graphically in Fig. 1).

These results show that the bubble model is a useful source of information for the prediction of TSS. The model, using probabilities for a bubble size (*m*) of 8, without any other kind of information, achieved the best results of 0.8083 for *G*-mean (at temperature 360K) and 0.8603 for auROC (at temperature 345K). These values are not far from the values obtained with the sequence. After we tested the usefulness of the bubble model, we considered the possibility of combining both sources of information (the sequence and the bubble model) using an SVM with a hybrid kernel [52]. We considered a hybrid kernel (see Equation 19) formed by a string kernel for the sequence part and a Gaussian kernel for the bubble model part. The linear combination (with the same weight, that is, the average) of both sources of information achieved a *G*-mean value of 0.8590 (at temperature 345K) and auROC of 0.9184 (also at temperature 345K), significantly improving the results obtained using the sequence alone. This result supported our hypothesis, as it showed that the information contained in the model is complementary to the information contained in the sequence.

**Fig. 2:**
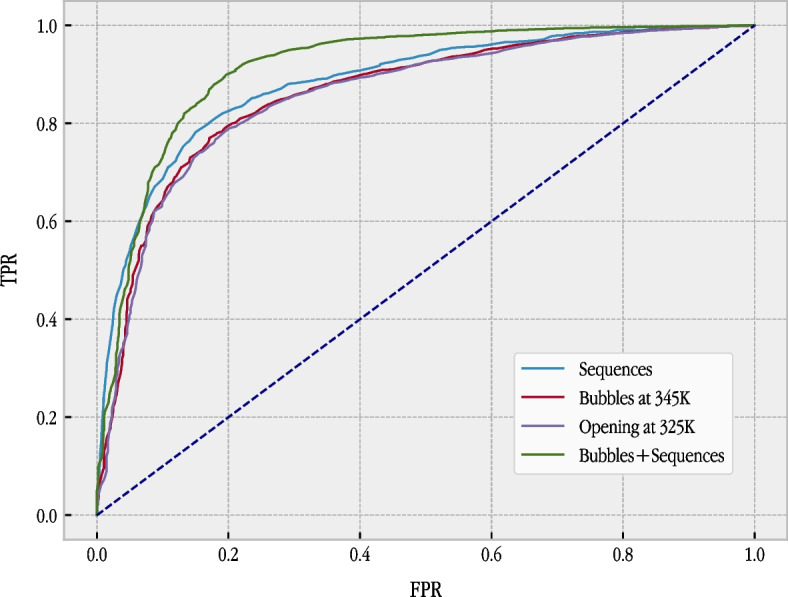
ROC curve for the best model for the standard approach using the sequence, bubbles, opening and the combination of bubble and sequences

These results are illustrated in Fig. 2. ROC curves for the standard method using the DNA sequence, bubbles at 345K, opening at 325K and the combination of the sequences and bubbles at 345K are shown. The plot shows that bubbles and openings were able to almost match the performance of the best current method of using sequences and a string kernel. Combining this source of information with the information given by the bubble model improved the results, with a ROC curve consistently better than that achieved by the standard method.

## Discussion

As previous works [3, 5, 11, 27] suggested, the separation of complementary bases in DNA strands, the DNA bubbles, influence biological processes. Therefore, physical models that predict the probability of these bubbles occurring can be a useful tool in the study of these biological processes. In this work, we used a well-known mesoscopic model of DNA: the PBD model. The results obtained in laboratory experiments provided evidence of the validity of this model [3, 4, 11]. The results of this work reinforce this evidence, but instead of using laboratory approaches, measuring deutorium-proton exchanges, using radioactive markers, or measuring UV absorption, machine learning models were used. On the one hand, it has been shown that ML models using only bubble probabilities provide competitive results in predicting TSS. On the other hand, it is possible to obtain better models when, in addition to the sequence of bases, the bubble information is added to the training step. Both facts are a strong indication that the bubble probabilities obtained by the PBD model provide relevant information about the biological processes of transcription initiation, since they help to improve the predictions of the ML models.

In addition to the results for the physical model, this work contributes to the fields of bioinformatics and machine learning in showing how fusing two views of DNA sequences can improve the performance of the obtained machine learning models.

The improved prediction capability of the TSS suggests that incorporating bubble probabilities can also improve the detection of other interesting protein binding sites in the DNA sequence. This is a line of research we will explore in the future. It will also be interesting to investigate whether all the improvements and extensions proposed for the initial PBD model [3] allow even better machine learning models to be created.

## Methods

### PBD model

The PBD model reduces the myriad degrees of freedom to a one-dimensional chain of effective atom compounds, describing the relative separation of the base pair from the positions of the ground state. This model [9] can be viewed as a mesoscopic dynamic model of the DNA molecule that ignores its helicoidal structure and condenses all atomic coordinates of a base pair into a single number *y* that describes the stretching of the bonds between the two bases. It attempts to complete the Ising model approach for DNA because it not only considers the closed and open states of a base pair, but also all intermediate states. This allows a description of the dynamics of the base pair fluctuations. Thus, additional information is provided to validate the model and calibrate its parameters. The basic model is defined by its Hamiltonian:

1 $$\begin{aligned} H\! =\! \sum _n \frac{p_n^2}{2m}\! +\! W(y_n,y_{n-1})\! +\! V(y_n), \text { with } p_n\! =\! m \frac{dy_n}{dt} \end{aligned}$$

**Fig. 3:**
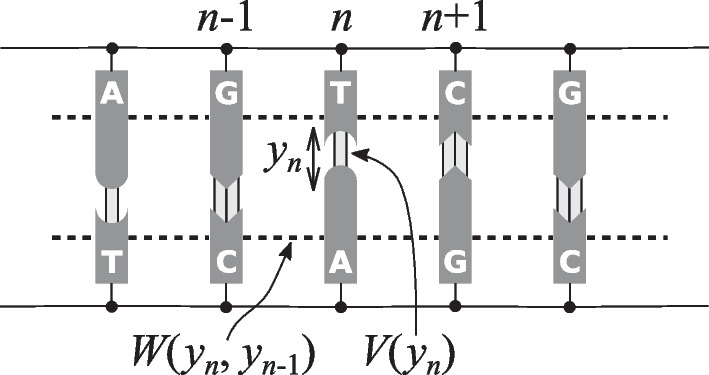
Simple dynamic model for DNA nonlinear dynamics as described by the Hamiltonian (Equation 1)

where *n* is the index of a base pair and *m* its reduced mass (the interpretation of all these terms is visually shown in Fig. 3). In this work, the total potential energy for a *N* base pair DNA chain is given by $V_1(y_1)+\sum _{i=2}^N V_i(y_i) + W(y_i,y_{i-1})$ with

2 $$\begin{aligned} V_i(y_i)= & {} D_i \Big ( e^{-a_i y_i}-1\Big )^2 \nonumber \\ W(y_i,y_{i-1})= & {} \frac{1}{2} K \Big ( 1+\rho e^{-\delta (y_i+y_{i-1})}\Big )(y_i - y_{i-1})^2 \end{aligned}$$

The first term, $V_i$, is the *on-site Morse potential* that describes the hydrogen bond interaction between bases on opposite strands. $D_i$ and $a_i$ determine the depth and width of the Morse potential, respectively, and are different for the AT and GC base pair. The *stacking potential* *W* consists of harmonic and nonlinear terms. The second term was introduced later [26] and mimics the effect of decreasing overlap between $\pi$ *electrons* when one of two neighbouring base moves out of the stack. As a result, the effective coupling constant of the stacking interaction drops from $K'=K(1+\rho )$ to $K'=K$. It is due to this term that the sharp phase transition that has been observed in denaturation experiments can be reproduced.

As described by Peyrard and Cuesta-López [11], entropic effects must be taken into account in this local potential $V(y_n)$ as well as in the coupling potential *W* because, irrespective of the stacking interaction, when a base flips out of the DNA stack, it gains new degrees of freedom (such as a possible rotation of the plane of the base plateau) that were constrained in the helical structure. However, in a mesoscopic model such as the one that we are considering, the “potentials” are actually potentials of mean force, which take into account all other degrees of freedom that are ignored in the model, in a statistical way. The entropy gain that follows the flipping of a single base out of the stack lowers the effective potential. In order to close again, the base has to overcome a very large entropic barrier. For the correct dynamics of the open states, this entropic barrier must be included in the potential *V*(*y*). The existence of this barrier was observed in free energy calculations deduced from all-atom molecular dynamics simulations of DNA [53]. In addition to the entropic effect, a barrier for closing might have a pure enthalpy contribution [54, 55] because the open bases tend to form hydrogen bonds with the solvent, which must be broken before closing. We have chosen the expression as described in [11]:

3 $$\begin{aligned} V(y) = D \left( e^{-\alpha y} - 1 \right) ^2 + \Theta (y) \frac{b y^3}{\cosh ^2[ c(\alpha y - d \; \ln 2)]} \end{aligned}$$

where $\Theta (y)$ is the Heaviside step function, which ensures that the term added to the Morse potential only plays a role for $y>0$. This expression was chosen because it has the correct qualitative shape to generate the entropic barrier and, due to the factor $y^3$, the frequency at the bottom of the potential is not affected by the additional contribution. The parameter *b* determines the amplitude of the barrier, *c* its width, and *d* its position in units of the value of *y* at the inflection point of the Morse potential. These are constants for a given type of base pair.

All interactions with the solvent and ions are effectively included in the force field. The constants ${K, \rho ,\alpha , D_{\text{AT}}, D_{\text{GC}}}$ and ${a_\text{AT}, a_{\text{GC}}}$ were parameterized in [56] and tested on denaturation curves of short heterogeneous DNA segments.

The orientation of the strands provides 16 possibilities for the stacking potential *W*. Considering all possibilities immediately introduces a large number of parameters in the model because the expression of the potential *W* depends on three parameters: the strength of the interaction *K* (having the dimension of an energy divided by the square of a length); the magnitude $\rho$ (dimensionless) of the variation of the stacking when base pairs open because the effective constant drops from $K(1 + \rho )$ to *K* when at least one of the interacting base pairs opens; and $\delta$ (dimension of the inverse of a length), which determines the size of the opening of a base pair for this variation to play a role. To reduce the number of parameters, we selected the same value of $\rho$ and $\delta$ for all stacking interactions. We set $\rho = 25$, which produces a large decrease in the stacking when either of the interacting base pairs opens. This is a necessary condition to have a sharp denaturation transition in DNA [57], in agreement with the experiments. A large value of $\rho$ is necessary to match neutron scattering experiments that probe the length of the closed regions of DNA versus temperature [58]. The value $\delta = 0.8$ was chosen because it leads to a decrease in the factor $\exp (-\delta y)$ to $\frac{1}{5}$ of its original value for a stretching $y \approx 2\;$Å of a base pair. This corresponds to overcoming the barrier in the intra pair potential *V*.

**Table 3:** Potential parameters used in the PBD model

Potential *V*	*D*	$\alpha$	*b*	*c*	*d*	
AT base pair	$0.09075\;$eV	$3.0\;$Å$^{-1}$	$4.00\;$eV	$0.74\;$Å$^{-1}$	$0.20\;$	
GC base pair	$0.09900\;$eV	$3.4\;$Å$^{-1}$	$6.00\;$eV	$0.74\;$Å$^{-1}$	$0.20\;$	

Potential *W*	$\rho$			$\delta$		
	25			0.8Å$^{-1}$		

Dimer	A–T	A–A	T–T	G–T	A–C	T–A
$K\;$(eV Å$^{-3}$)	0.00176	0.00418	0.00418	0.00480	0.00462	0.00506

Dimer	G–A	T–C	C–C	G–G	G–C	C–T
$K\;$(eV Å$^{-3}$)	0.00546	0.00546	0.00810	0.00810	0.00865	0.00865

Dimer	A–G	C–A	T–G	C–G		
$K\;$(eV Å$^{-3}$)	0.00865	0.01140	0.01140	0.01690		

Consequently, the dependence of the sequence in the interaction is entirely included in the variation of *K*. *K* values for the stacking has been defined accordingly to the differences in the ‘pi’ stacking energies resulting from differences arising from rise, twist, and slide computed from quantum calculations derived from geometries extracted from a collection of B-DNA structures [59]. We selected a set of parameters based on the results of theoretical calculations of stacking energies [60] and evaluations of the stabilities of DNA doublets deduced from thermodynamic measurements. Furthermore, the values for the model [11] have been selected in a compromise to reproduce both the melting of short sequences as in [61]. The selection of the stacking values is in agreement with the prediction of the melting temperatures of the PBD model defined in [11] for the different cases of homopolymer. Differences in stacking values have been considered after analysing results of quantum chemistry calculations as reported in [62]. Table 3 lists all parameters that we selected for the model.

These examples show that despite its simplified character, the model can provide a quantitative description of DNA. Most importantly, it allows us to study the statistical and dynamic behaviour of very long heterogeneous DNA sequences, which is impossible in any atomistic model.

The PBD model basically represents a single dsDNA in an infinite solution. The probability for the denatured state tends to unity with increasing time at any temperature. It is, therefore, only in the limit of infinite long chains that denaturation curves can be reproduced without additional assumptions.

An exact theoretical calculation of the model and the partition function governing the statistics for open basis probabilities can be provided since the model is one-dimensional. For a DNA molecule that comprises *N* base pairs, it reduces to a sequence of one-dimensional integrals over the variables $y_1 \ldots y_N$. This can be performed by a simple iterative scheme, and all the details that we have followed have reproduced the methodologies of Prof. M. Peyrard [11] and Prof. N. Theodorakopoulos [63].

Let us give a sketch of the process (for a complete discussion, see [11] and [63]). Let:

4 $$\begin{aligned} Z_I = \int \Pi dy^N e^{-\beta U(y^N)} \end{aligned}$$

be the configuration integral performed over an infinite domain for each $y_i$. In practice, the calculation has to be done over a finite domain with an appropriate cut-off [10]), where $y^N$ denotes the ensemble of all variables $y_1 \ldots y_N$, and $U(y^N)$ is the potential energy of the model, the sum of the *W* and *V* contributions. Similarly, we can define

5 $$\begin{aligned} Z_{ II } = \int _{y^N > \xi } \Pi dy^N e^{-\beta U(y^N)} \end{aligned}$$

to be the same integral with the condition that *all* the $y_i$ are simultaneously larger than $\xi$. $Z_{ II }$ gives the statistical weight of the fully open states of the model so that the configuration integral of the dsDNA ensemble is

6 $$\begin{aligned} Z = Z_I - Z_{ II } \; , \end{aligned}$$

which is well-defined and does not depend on the upper cut-off. To obtain, for instance, the statistical weight of the states for which the base pair *j* is closed, we have to compute

7 $$\begin{aligned} Z (j \;\text {closed}) = \int _{y_j < \xi } \Pi dy^N e^{-\beta U(y^N)} \end{aligned}$$

where $y_j$ is constrained to be smaller than $\xi$, with the others unconstrained. This gives the probability that the base pair *j* is closed in the dsDNA ensemble as

8 $$\begin{aligned} P_\text {dsDNA}(j \; \text {closed}) = \frac{Z (j \; {\text{closed}})}{ Z_I - Z_{ II }} \; , \end{aligned}$$

Thus, we can compute the probability that each single base pair is open as a function of temperature.

In addition to denaturation curves, and single base pair opening probabilities, the statistical method introduced in [11, 50, 63] allows studying bubbles of a given size.

Consequently, we can define the bubble probability matrix ${P_{\text{bub}}(k,m)}$ as the probability to have a bubble of size *m* centred on the base pair *k* provided that the molecule is part of the dsDNAE. Therefore,

9 $$\begin{aligned} P_{\text{bub}}(k,m) \equiv \left\langle \theta _k^{[m]}\right\rangle _\mu {\quad \text { with } \quad \mu =1-\prod _{i=1}^N \theta _i} \end{aligned}$$

Being, $\mu =1$, except when all bases are open, in which case $\mu =0$.

In principle, ${P_{\text{bub}}(k,m)}$ contains all the information on bubble statistics in a DNA sequence. It is also useful to calculate other quantities. From a physical and biological perspective, it might be useful to know the ability to participate in bubbles. Therefore, we introduce the ${P_{\text{part}}(k,m)}$ probability, which is the probability of participating in a bubble of at least *m* sites.

10 $$\begin{aligned} P_{\text{part}}(k,m)\equiv & {} \!\!\sum _{m' \ge m}^{\{ m' \text {: even}\}} \, \, \sum _{k'=k-m'/2}^{k+m'/2-1}\!\! P_{\text{bub}}(k',m') \nonumber \\{} & {} \quad + \!\!\sum _{m' \ge m}^{\{ m' \text {: odd}\}} \sum _{k'=k-(m'-1)/2}^{k+(m'-1)/2}\!\!\!\!\! P_{\text{bub}}(k',m') \end{aligned}$$

This quantity is less mathematically stringent, as it is independent of where you assign the position of the bubble. Note that this quantity is still somewhat different from the projection in [50] where each bubble is still associated with one base pair position only. In variance with ${P_{\text{bub}}(k,1)}$, the bubble participation probability ${P_{\text{part}}(k,1)}$ is directly related to the simple opening. Hence, ${P_{\text{part}}(k,1)= \left\langle \theta _k \right\rangle _\mu \ne P_{\text{bub}}(k,1)}$.

Defining the probabilities by means of a set of functions:

11 $$\begin{aligned} \theta _i(y_i)=\theta (y_i-y_0), \qquad {\bar{\theta }}_i(y_i)=\theta (y_0-y_i) \end{aligned}$$

where $\theta (\cdot )$ is the Heaviside step function. $\theta _i$ equals 1 if the base pair is open and is zero otherwise. ${\bar{\theta }}_i$ is the reverse.

The value $y_0$ is an important threshold to define when a base pair is considered open or closed, as an equilibrium distance for the potential of the base pair.Higher values of $y_0$ will require higher temperatures to melt the double strand and to generate bubbles, and the cooperativeness of the bubbles will probably be affected^1^. The value 1.5 has been selected, which is the average base separation according to DNA melting experiments that examine the structure of DNA under thermal fluctuations [64] and it is also a value aligned with the parameters of the PBD model described in [11].

Thus:

12 $$\begin{aligned} \theta _i^{[m]}\equiv & {} {\bar{\theta }}_{i-\frac{m}{2}} {\bar{\theta }}_{i+\frac{m}{2 }+1} \prod _{j=i-\frac{m}{2}+1}^{i+\frac{m}{2}} \theta _j \text { for { m} even} \nonumber \\\equiv & {} {\bar{\theta }}_{i-\frac{m+1}{2}} {\bar{\theta }}_{i+\frac{m+1}{2}} \prod _{j =i-\frac{m-1}{2}}^{i+\frac{m-1}{2}} \theta _j \text { for { m} odd} \end{aligned}$$

which are 1 (0 otherwise) if and only if *i* is at the centre of a bubble that has the exact size *m*. To shorten the notation, we removed the $y_i$ dependencies. For even numbers, it is a bit arbitrary where to place the centre, but we defined it as the base directly to the left of the midpoint of the bubble. To also have these quantities defined near the ends of the chain, we use ${\bar{\theta }}_i=1$ for $i = 0$ and $i=N+1$. The properties of interest are the probabilities of bubbles of size *m* centred on base pair *i*, provided that the molecule has a double-strand configuration.

13 $$\begin{aligned} \left\langle \theta _i^{[m]}\right\rangle _\mu\equiv & {} \frac{\left\langle \theta _i^{[m]} \mu \right\rangle }{\left\langle \mu \right\rangle } \end{aligned}$$

14 $$\begin{aligned}\equiv & {} \frac{Z_{\theta _i^{[m]}} }{Z-Z_\Pi } \end{aligned}$$

Thanks to the formalism described above, the partition function integrals can be easily defined and are given by

$$\begin{aligned} Z&= \int {\mathrm d} y^N e^{-\beta \Big [ V_N(y_N)+W(y_N,y_{N-1})+ \ldots +W(y_2,y_1)+V_1(y_1) \Big ]} \\ Z_{\theta _i^{[m]}}&= \int {\mathrm d} y^N e^{-\beta \Big [ V_N(y_N)+W(y_N,y_{N-1})+ \ldots +V_1(y_1) \Big ]} \theta _i^{[m]} \\ Z_\Pi&= \int {\mathrm d} y^N e^{-\beta \Big [ V_N(y_N)+W(y_N,y_{N-1})+ \ldots +V_1(y_1) \Big ]} \times \prod _j \theta _j. \end{aligned}$$

The partition functions described above can be exactly integrated following the procedures described in [10, 11, 63].

**Fig. 4:**
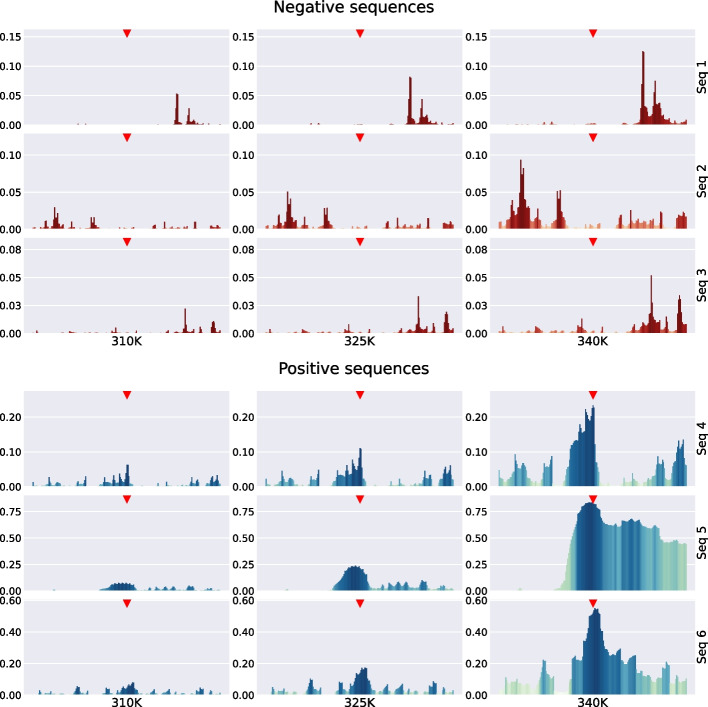
Examples of probability sequences for openings ($P_\text {part}(k,1)$ as a function of *k*) . In each subfigure, the top red triangle at the centre of the sequence marks the point that would correspond to TSS. The length of the bar represents opening probability. Top subfigure is for negative examples, sequences that do not correspond to TSS (see the specific sequences in Fig. 6). The bottom subfigure corresponds to positive examples, sequences for which TSS occurs at the centre of the sequence (see the specific sequences in Fig. 7)

**Fig. 5:**
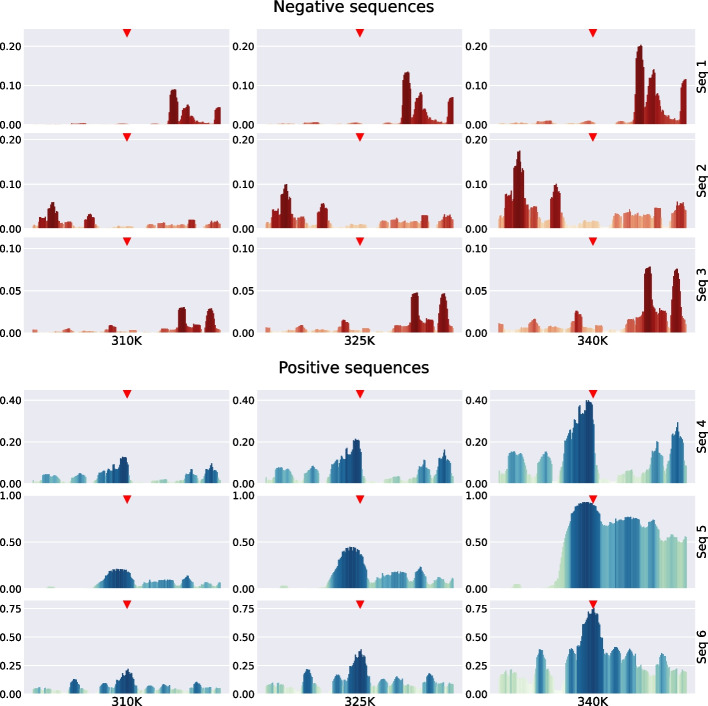
Examples of probability sequences for bubbles of size 8 ($P_\text {part}(k,8)$ as a function of *k*). In each subfigure, the top red triangle at the centre of the sequence marks the point that would correspond to TSS. The length of the bar represents opening probability. The top subfigure is for negative examples, sequences that do not correspond to TSS (see the specific sequences in Fig. 6). The bottom subfigure corresponds to positive examples, sequences for which TSS occurs at the centre of the sequence (see the specific sequences in Fig. 7)

**Fig. 6:**
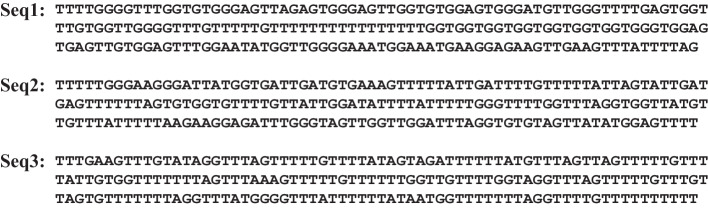
Negative sequences used in Figs. 4 and 5

**Fig. 7:**
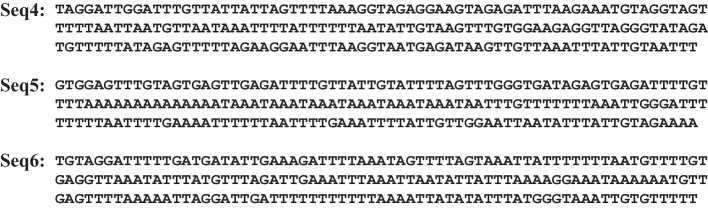
Positive sequences used in Figs. 4 and 5

Using the procedure outlined, the PBD model can be used to transform a sequence of DNA bases into a sequence of probabilities for which an opening or bubble occurs at the position of each base. These probabilities can be used as input features to obtain relatively simple TSS prediction models with results on the same level as the state-of-the-art proposals, including some very sophisticated and complex that combine several input features to accomplish the same task [34, 65–67]. Figure 4 shows several probability profiles for opening probabilities (that is, the probabilities that there is a bubble of size 1, at a particular base *k*, $P_\text {part}(k,1)$) for the bases of several sequences at different temperatures (three sequences of negative TSS cases and three sequences corresponding to positive TSS cases). Similarly, Fig. 5 shows the probabilities of bubbles of size 8, $P_\text {part}(k,8)$, centred on the bases of the same six sequences.

### SVM and string kernels

In their most basic use, support vector machines (SVMs) [68] obtain models able to determine if an instance of a dataset $S = \{(x_1, y_i), \ldots , (x_N,y_N)\}$ belongs to one of two possible classes (that is $y_i\in \{-1, 1\}$). This type of problem is known as *binary classification* (although there have been later developments that allow their use in general classification problems with several classes and regression problems where instead of a categorical value, the prediction is a continuous value). Focussing on binary classification, SVMs seek to find the “best” hyperplane that separates the two classes in a dataset. Assuming that the two classes are linearly separable, the “best” hyperplane is the one whose distance to the closest instance of each class is the greatest possible or, in SVM terminology, the hyperplane that maximises the “margin.” Formally, this can be expressed as a maximisation problem:

$$\begin{aligned} \begin{aligned} \max _{{\textbf{w}}, b}&\quad \frac{1}{\Vert {\textbf{w}}\Vert } \\ \text {subject to}&\quad \min _{i=1, 2, \ldots , N} |{\textbf{w}}^T{\textbf{x}}_i+b|= 1 \\ \end{aligned} \end{aligned}$$

where ${\textbf{w}}$ is restricted so that for the closest point ${\textbf{x}}_c$ to the plane $|{\textbf{w}}^T{\textbf{x}}_c+b|= 1$ (the *canonical hyperplane*). The difficulties posed by the restrictions as they appear in this initial statement of the problem are solved by transforming it into a minimization such as

15 $$\begin{aligned} \begin{aligned} \min _{{\textbf{v}}, b, \xi } \quad&\frac{1}{2}{\textbf{v}}^{T}{\textbf{v}}+C\sum _{i=1}^{N}{\xi _{i}}\\ \text {s.t.} \quad&y_{i}\big ({\textbf{v}}^T\Phi ({\textbf{x}}_{i})+b\big ) \ge 1-\xi _{i},\ \ i=1, \ldots , N\\&\xi _i\ge 0,\ \ i=1, \ldots , N \end{aligned} \end{aligned}$$

where, in addition, the approach has been generalised so that the hyperplane allows the division of the two classes in a space of higher dimensionality (possibly infinite) to which the training vectors have been mapped with the function $\Phi$ (note that now ${\textbf{v}}$ is the vector defining the hyperplane in the feature space). It is also taken into account that we may be unable to prevent some instance from being on the wrong side of the hyperplane (the influence of these misplaced instances is controlled by the parameter *C*). This is the *soft margin formulation* that increases the power of SVMs to find optimal decision boundaries even though the starting space is not linearly separable or when a perfect separation is not possible.

In practise, it is easier to solve the dual problem:

16 $$\begin{aligned} \begin{aligned} \max _{\mathbf {\alpha }} \quad&\sum _{i=1}^N-\frac{1}{2}\sum _{i=1}^N\sum _{j=1}^N \alpha _i\alpha _jy_iy_j\Phi ({\textbf{x}}_i)^T\Phi ({\textbf{x}}_j)\\ \text {s.t.} \quad&0\le \alpha _i \le C, ,\ \ i=1, \ldots , N\\&\sum _{i=1}^N\alpha _iy_i = 0 \\&\alpha _i\ge 0,\ \ i=1, \ldots , N \end{aligned} \end{aligned}$$

whose solution has the particularity that most of the $\alpha _i$ values are zeros. When $\alpha _i$ is nonzero, the corresponding vector $\Phi ({\textbf{x}}_i)$ is called *support vector*. The decision function can be constructed using only the support vectors:

17 $$\begin{aligned} h({\textbf{x}}) = \text {sign}\left( \sum _{\alpha _j>0} \alpha _jy_j\Phi ({\textbf{x}}_j)^T\Phi ({\textbf{x}}) + b\right) \end{aligned}$$

where $b = y_k - \sum _{\alpha _j>0} \alpha _jy_j\Phi ({\textbf{x}}_j)^T\Phi ({\textbf{x}}_k)$ for any support vector ${\textbf{x}}_k$ with $0<\alpha _k<C$.

The other important aspect of SVMs is that, by means of the so-called *kernel trick*, it is not necessary to explicitly know the function $\Phi$. Rather, it is sufficient to have a function, the *kernel function* (sometimes called just *the kernel*), that allows us to calculate the dot product of the vectors in the transformed space, $K({\textbf{x}}_i, {\textbf{x}}_j) = \Phi ({\textbf{x}}_i)^T\Phi ({\textbf{x}}_j)$. There are a wide variety of kernel functions that are used in practise, each more suitable for some problems than others, including

- Linear: $$K({\textbf{x}}_i, {\textbf{x}}_j) = {\textbf{x}}_i^T\cdot {\textbf{x}}_j$$.
- Polynomial: $$K({\textbf{x}}_i, {\textbf{x}}_j) = (\gamma {\textbf{x}}_i^T\cdot {\textbf{x}}_j+r)^d, \gamma >0$$.
- Radial Basic Function (RBF): $K({\textbf{x}}_i, {\textbf{x}}_j) = \exp (-\gamma \Vert {\textbf{x}}_i-{\textbf{x}}_j\Vert ^2), \gamma >0$.

The kernel trick can also be used for strings (and sequences). We do not need to explicitly calculate the mapping $\Phi$ that transforms a sequence into a vector in a feature space. It is sufficient to know a kernel that calculates the dot product of the vectors in the feature space associated with the sequences. To be useful, these kernels should be mathematically valid, fast to compute and adapted to the problem. These kernels can be thought of as functions that measure the similarity of sequences.

The general approach to use SVM with sequences of symbols (from an alphabet ${\mathcal {A}}$) is to define a mapping $\Phi : {\mathcal {A}}^L \rightarrow {\mathbb {R}}^{{\mathcal {A}}^L}$ while determining an efficient way to calculate the kernel function that avoids having to explicitly compute the (high-dimensional) feature vector. The proposals differ in the way this mapping is accomplished and in how to efficiently calculate the kernel function. Some examples are

- *Spectrum kernel* [41]: for a string ${\textbf{s}}$, the feature space for this kernel is a histogram of the occurrences in ${\textbf{s}}$ of each possible substring *u* of size *k* over the alphabet ${\mathcal {A}}$ of nucleotides (these substrings are called *k*-*mers*). Although the size of the feature vectors is exponential in the length of the substrings, they are sparse, and the kernel can be calculated efficiently without obtaining them explicitly.
- *Mismatch kernel* [43]: this kernel also takes into account the appearance of the *k*-mers *u* in ${\textbf{s}}$ but in a more flexible way that allows up to *m* mismatches (without gaps).
- *Substring kernel* [42]: this kernel allows the matching of substrings *u* in ${\textbf{s}}$ to be with gaps, using an exponential decaying weight that penalises large gaps. The kernel can be calculated in polynomial time using dynamic programming.
- WD kernel (*Weighted Degree kernel*) [44]: with this kernel, the similarity is no longer given by the histogram of substrings in each sequence but by a direct comparison between the sequences, counting the (exact) co-occurrences of *k*-mers in the two sequences. To compare two sequences ${\textbf{s}}_i$ and ${\textbf{s}}_j$ (both of length *L*), the WD kernel of order *d* uses a weighted sum of all contributions of *k*-mer matches of lengths $k \in \{1, \ldots , d\}$:
  18 $$\begin{aligned} k({\textbf{s}}_i, {\textbf{s}}_j) = \sum _{k=1}^d \beta _k \sum _{l=1}^{L-k+1} {\mathbb {I}}\big ({\textbf{u}}_{k, l} ({\textbf{s}}_i) = {\textbf{u}}_{k, l} ({\textbf{s}}_j)\big ) \end{aligned}$$
  ${\textbf{u}}_{k,l}({\textbf{s}})$ is the oligomer of length *k* starting at position *l* of the sequence ${\textbf{s}}$, and ${\mathbb {I}}(\cdot )$ is an indicator function that returns 1 when its argument is true and $\beta _k = 2(d-k+1)/(d(d+1))$. Note that even though $\beta _{k+1}<\beta _k$, long matches contribute more to Equation 18 because they add the contribution of the short matches that they include.
- WDS kernel (*Weighted Degree kernel with Shifts kernel*) [44]: this kernel represents an intermediate situation between the WD kernel, which is totally rigid as to the position in which matches occur, and the spectrum kernel, for which positional information is not relevant. It is sufficient that sequences share substrings regardless of the position in which they occur. WDS is defined as
  $$\begin{aligned} k({\textbf{s}}_i, {\textbf{s}}_j) = \sum _{k=1}^d \beta _k \sum _{l=1}^{L-k+1} \gamma _l \sum _{\begin{array}{c} s=0\\ s+l\le L \end{array}}^{S(l)} \frac{1}{2(s + 1)} \\ \Big ( {\mathbb {I}}\big ({\textbf{u}}_{k, l+s} ({\textbf{s}}_i) = {\textbf{u}}_{k, l} ({\textbf{s}}_j) \big ) + {\mathbb {I}}\big ({\textbf{u}}_{k, l} ({\textbf{s}}_i) = {\textbf{u}}_{k, l+s} ({\textbf{s}}_j) \big )\Big ) \end{aligned}$$
  which now has three weights: one for the *k*-mers, $\beta _j$, with the same definition as in WD; $\gamma _l$ for the positions in which the *k*-mers can appear in the sequence; and $1/(2(s+1))$ associated with the shifts, whose range *S*(*l*) is different for each position *l*.

To expand the toolbox of available kernels, several can be combined to obtain new kernels that can integrate different notions of similarity by combining multiple information sources. The combination itself can be learned (multiple kernel learning, or MKL), so now the kernel is

19 $$\begin{aligned} k_\eta ({\textbf{x}}_i, {\textbf{x}}_j) = f_\eta \Big (\big \{k_m({\textbf{x}}^m_i, {\textbf{x}}^m_j)\big \}_{m=1}^P\Big ) \end{aligned}$$

where $f_\eta$ is a combination function that can be linear or nonlinear with domain ${\mathbb {R}}^P$ and range ${\mathbb {R}}$. Kernel functions $\{k_m:{\mathbb {R}}^{D_m}\times {\mathbb {R}}^{D_m}\rightarrow {\mathbb {R}}\}_{m=1}^P$, take *P* features representations (not necessarily different) of data instances: ${\textbf{x}}_i=\{{\textbf{x}}_i^m\}_{m=1}^P$ where ${\textbf{x}}_i\in {\mathbb {R}}^{D_m}$, and $D_m$ is the dimensionality of the corresponding feature representation [52].

## Data Availability

The code for the ML models and the sequence data is publicly available at http://cib.uco.es/nonlinear-physics/. The simulation code for the physical DNA model will be available upon reasonable request by email to the second author.

## Footnotes

1 It is interesting to note that the effect on the output of the ML model of variations in the value $y_0$ is relatively equivalent to the effect of varying the simulation temperature. Therefore, the results of the experiments for different temperatures are a good proxy of the effect of the parameter $y_0$ on the ML model.
